# Metabolism in Health and in Disease

**Published:** 1907-06-01

**Authors:** 


The Hospital

A Journal of

The Medical Sciences and Hospital Administration.

New Series. No. 9, Vol. I. [No. 1081, Vol. XLII.] SATURDAY, JUNE 1, 1907.

### METABOLISM IN HEALTH AND IN DISEASE.

Metabolism—that is to say, the chemical changes that occur in the food from the time it is ingested until its end-products are eliminated from the system—lies so absolutely at the root of all life, whether healthy or diseased, that no medical man can afford not to know as much about it as is possible. It does not follow, of course, that because the metabolism of a particular man or woman is wrong that the best thing to do is to take steps to alter it. In many cases the patient's metabolic processes, wrong though they are, are the best that the patient's body is capable of under the circumstances; but to realise whether this is so or not, the patient's medical adviser must know all he can about metabolism, or else he will not know what should be left alone, and what would be the better of modification by some dietetic, medicinal, or other means to hand. It is further necessary to know a great deal about metabolism to prevent the development of faddisms in diet rules. Faddists, as regard diet, are nearly always those who know one aspect of metabolism only; such faddists upon the subjects of uric acid metabolism, or of intestinal toxæmia, to take two examples, are too common; they would be less common if they had a broader view of metabolism, and they would learn that the important things are: not *what* you eat, but *when* you eat, *how* you eat, and *how much* you eat. There are very few foodstuffs which cannot be properly metabolised in some degree by everyone; it is the amount which should be taken which needs modification. Even in diabetes mellitus, for example, a certain amount of carbohydrate should be allowed to every patient to get the best result, but the best result will be obtained with less carbohydrate in one case of diabetes than in another.

Views upon the question of meat diet in renal cases have undergone immense changes of late years, and the reasons for these changes depend upon the results of metabolism research. We have not space to elaborate the subject, but it must be clear to all that metabolism in disease, in its differences from metabolism in health, is a most important branch of medical study.

The main trouble is that metabolic studies, both in physiology and in pathology, are of comparatively recent date, and the papers upon the subject are scattered far and wide throughout the literature of Europe and America. The text-books of physiology have collected together the main points about metabolism in healthy persons and animals, but hitherto there has been a great need for a similar action in regard to metabolism in disease. This need has been supplied by Professor Von Noorden and his collaborators in the book which we review upon page 235. All the various papers bearing upon the matter have been carefully collected, and, what is more important still, they have been sifted, and the good picked out from amongst the bad. This sifting is a most laborious process, for so many indifferent papers are printed nowadays; even now we are bound to say that much is left which will need a further sifting in years to come. The busy practitioner will be apt to say that he cannot find his way amongst all the pros and cons that are adduced for each small point that is argued in the book; nevertheless, it is only by studying the pros and cons that the value of particular views can be arrived at, and to few questions in metabolism can the answers be dogmatic.

There are many side issues arising out of research work of any kind; chemical pathologists, in their endeavour to throw light upon the causation of diseases, have again and again discovered points which are of the greatest value to clinical physicians and surgeons. It is to recent researches upon metabolism that we owe our knowledge of the importance of diacetic acid, acetone, and oxy-butyric acid in the urine in cases of diabetes; the relation of these substances to the incidence of coma; and the prophylactic measures, such as the administration of sodium bicarbonate, which may be adopted to ward off the coma. It is to metabolism work that we owe the many different methods we now have of diagnosing pancreatic diseases with much greater accuracy than formerly—by chemical examination of the fæces, for example, or by the administration of keratin-coated capsules, which are digested only when the pancreatic juice is active, and so on. The inability of the diseased kidneys to excrete sodium chloride properly has been known for years in Germany, though it has only recently been recognised in England; the side issue of this metabolic fact is its immediate bearing upon the dropsy of renal cases and the power we have to check or limit this dropsy by modifying the sodium chloride ingested by these patients. Theories upon intestinal toxæmia have shown a tendency to run riot of late years, but unbiassed workers in the laboratories have been investigating the matter, and it has become increasingly clear that this toxæmia is by no means proved even to exist at all, still less to be the cause of all the troubles that have been attributed to it. It is most useful to have chemical data for verifying or refuting bedside theories, either of pathology or of treatment, and it is essential that the medical man should try to keep abreast of the main results that are being found. For some years past pathology in England has been almost synonymous with bacteriology, but pathology has other aspects also, and the least known of these is the chemical or metabolic aspect. We are very fortunate in now having a really good book upon the subject in the English language, albeit a translation from the German.

